# ADAR2-dependent RNA editing of GluR2 is involved in thiamine deficiency-induced alteration of calcium dynamics

**DOI:** 10.1186/1750-1326-5-54

**Published:** 2010-11-27

**Authors:** Shuchen Lee, Guang Yang, Yue Yong, Ying Liu, Liyun Zhao, Jing Xu, Xiaomin Zhang, Yanjie Wan, Chun Feng, Zhiqin Fan, Yong Liu, Jia Luo, Zun-Ji Ke

**Affiliations:** 1 Key Laboratory of Nutrition and Metabolism, Institute for Nutritional Sciences, Shanghai Institutes for Biological Sciences, Chinese Academy of Sciences, Shanghai 200031, China; 2 Department of Anesthesiology, Gongli Hospital, Pudong, Shanghai, China; 3 Department of Internal Medicine, University of Kentucky College of Medicine, 130 Bosomworth Health Science Research Building, 1095 Veterans Drive, Lexington, Kentucky 40536, USA

## Abstract

**Background:**

Thiamine (vitamin B1) deficiency (TD) causes mild impairment of oxidative metabolism and region-selective neuronal loss in the central nervous system (CNS). TD in animals has been used to model aging-associated neurodegeneration in the brain. The mechanisms of TD-induced neuron death are complex, and it is likely multiple mechanisms interplay and contribute to the action of TD. In this study, we demonstrated that TD significantly increased intracellular calcium concentrations [Ca^2+^]_i _in cultured cortical neurons.

**Results:**

TD drastically potentiated AMPA-triggered calcium influx and inhibited pre-mRNA editing of GluR2, a Ca^2+^-permeable subtype of AMPA receptors. The Ca^2+ ^permeability of GluR2 is regulated by RNA editing at the Q/R site. Edited GluR2 (R) subunits form Ca^2+^-impermeable channels, whereas unedited GluR2 (Q) channels are permeable to Ca^2+ ^flow. TD inhibited Q/R editing of GluR2 and increased the ratio of unedited GluR2. The Q/R editing of GluR2 is mediated by adenosine deaminase acting on RNA 2 (ADAR2). TD selectively decreased ADAR2 expression and its self-editing ability without affecting ADAR1 in cultured neurons and in the brain tissue. Over-expression of ADAR2 reduced AMPA-mediated rise of [Ca^2+^]_i _and protected cortical neurons against TD-induced cytotoxicity, whereas down-regulation of ADAR2 increased AMPA-elicited Ca^2+ ^influx and exacerbated TD-induced death of cortical neurons.

**Conclusions:**

Our findings suggest that TD-induced neuronal damage may be mediated by the modulation of ADAR2-dependent RNA Editing of GluR2.

## Background

Thiamine (vitamin B1) deficiency (TD) induces chronic mild impairment of oxidative metabolism and causes neuroinflammation, leading to neuronal loss in specific brain regions [1]. Experimental TD causes a reduction of thiamine-dependent enzyme activities in multiple brain regions which is also observed in patients with Alzheimer's disease (AD) [2,3]. Since TD-induced neuronal damages and aging-associated neurodegeneration share many common features, TD in animals has been used to model the pathogenesis of aging-related neurodegeneration in humans. A recent study shows benfotiamine, a thiamine derivative with better bioavailability than thiamine, has powerful beneficial effects on cognitive impairment in the Morris water maze and β-amyloid deposition in amyloid precursor protein/presenilin-1 transgenic mice [4]. The TD in humans causes Wernicke-Korsakoff syndrome (WKS), which is characterized by severe memory loss, cholinergic deficits and selective cell death in specific brain regions [1,5-7]. The causes for TD-induced neuronal damage remain unclear. Several potential mechanisms have been proposed; these include mitochondrial dysfunction [8,9], impairment of oxidative metabolism [10,11] and acidosis [12,13]. We have recently demonstrated that TD causes endoplasmic reticulum (ER) stress in neurons, and ER stress may contribute to TD-induced neuronal damage [14]. ER stress is caused by the accumulation of unfolded proteins in the ER lumen which is often provoked by the inhibition of protein glycosylation and the perturbation of calcium homeostasis [15-17].

In the late stage of TD, an increase in extracellular glutamate is observed in some brain regions [6,18]. The selective vulnerability to TD may be mediated by a glutamate-induced excitotoxic process in affected structures, leading to alterations in membrane potential and disturbances in calcium homeostasis [19,20]. Calcium ions (Ca^2+^) can enter neurons through several mechanisms. One important mechanism is through the activation of glutamate receptors [21]. There are three types of ionotropic glutamate receptors: N-methyl-d-aspartate receptors (NMDARs), alpha-amino-3-hydroxyl-5-methyl-4- isoxazole-propionic acid receptors (AMPARs) and kainate receptors (KRs), each having several subtypes. The current study focuses on AMPARs. In the mammalian central nervous system (CNS), AMPARs are widely expressed both in neurons and in glia and mediate the vast majority of fast excitatory synaptic transmission [22,23]. AMPARs are tetramers made up of combinations of four subunits: GluR1, GluR2, GluR3 and GluR4 (also called ''GluRA-D'') [24,25].

The Ca^2+ ^permeability of AMPAR channels is determined by the GluR2 subunit [26-28]. The property of GluR2 is altered by pre-mRNA editing. This post-transcriptional modification involves the enzymatic deamination of a specific adenosine in the pre-mRNA prior to splicing [29]. The adenosine deamination results in the substitution of glutamine (Q) with arginine (R) in the membrane domain M2 of the receptor channel. The edited GluR2 (R) subunits form Ca^2+^-impermeable channels, whereas unedited GluR2 (Q) channels are permeable to Ca^2+ ^flow [29].

Enzymes responsible for RNA editing are termed "adenosine deaminases acting on RNA" (ADARs), and three structurally related ADARs (ADAR1 to ADAR3) have been identified in mammals [30-32]. ADAR1 and ADAR2 are widely detected in various tissues, with strong expression in the brain [30,33]. ADAR2 predominantly catalyzes RNA editing at the Q/R sites of GluR2 both *in vitro *and *in vivo *[34], whereas both ADAR1 and ADAR2 catalyze the Q/R sites of GluR5 and GluR6 subunits of kainite receptors. ADAR3 is detected only in the brain, but its deaminating activity has not been demonstrated [31,32]. ADAR2 pre-mRNA and mRNA themselves are susceptible to A-to-I editing mediated by ADAR2 [35]. The objective of the present study is to investigate the effect of TD on AMPAR-mediated Ca^2+ ^influx and GluR2 RNA editing. Our results show that TD down-regulates the expression of ADAR2 and inhibits GluR2 pre-mRNA editing at the Q/R site, resulting in increased Ca^2+ ^permeability. TD-induced disruption of Ca^2+ ^homeostasis may at least partially contribute to its neurotoxicity.

## Results

### Effects of thiamine deficiency (TD) on intracellular calcium concentration

**Figure 1:**
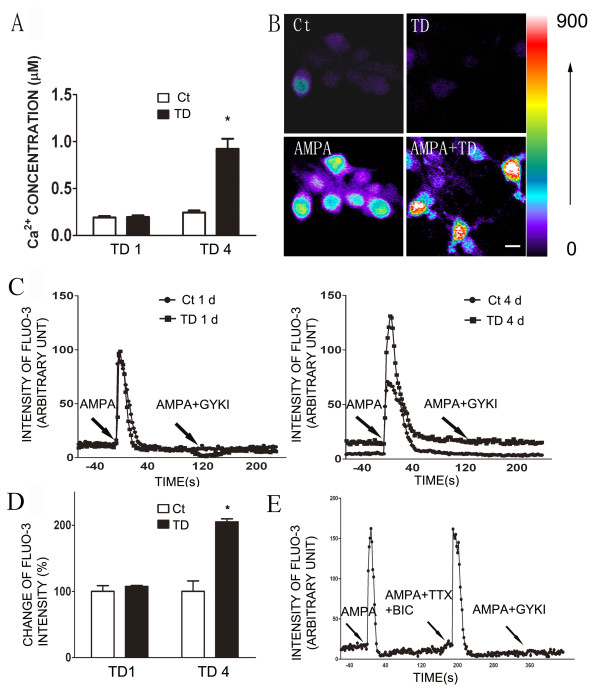
**The effect of TD on AMPA-stimulated [Ca^2+^]_i _in cultured cortical neurons**. **A**: Cortical neurons were maintained *in vitro *for 7 days (DIV7), and TD was induced by the treatment of amprolium (1 mmol/L) for 1 or 4 days (TD1 or TD4). The resting [Ca^2+^]_i _was measured as described under the Materials and Methods. **B**: Cortical neurons of DIV7 were treated with amprolium (0 or 1 mmol/L) for 4 days and perfused with AMPA (30 μM) for 5 sec. The calcium image (Fluo-3 fluorescence) was recorded as described under the Material and Methods. There were 240 cells for each treatment group. Scale bar = 20 μm. The colored scale bar indicates the fluorescence intensity of Fluo-3. **C**: Cortical neurons of DIV7 were treated with amprolium (0 or 1 mmol/L) for 1 or 4 days and then perfused with AMPA (30 μM) for 5 seconds. Two minutes after the first AMPA perfusion, neurons were subjected to a second AMPA perfusion with/without a general AMPAR antagonist GYKI (30 μM). Intracellular free levels [Ca^2+^]_i _were determined by single cell Fluo-3 fluorescence imaging. The data show a representative response to AMPA by a single cell. **D**: The intensity of Fluo-3 fluorescence was quantified as described under the Materials and Methods. The results were calculated based on 75 cells. **E**: Cortical neurons of DIV7 were treated with amprolium (1 mmol/L) for 4 days and perfused with AMPA (30 μM) for 5 sec, followed by a second perfusion with AMPA plus tetrodotoxin (TTX; 0.5 μM)/bicuculline (BIC; 10 μM). A third AMPA perfusion was performed in the presence of AMPAR antagonist GKKI (30 μM). The perfusions were 2 min apart. The results were expressed as the mean ± SEM. *p < 0.001. The experiments were replicated three times.

TD was induced in cortical neurons of DIV7 for one or four days as previously described [36]. Intracellular free calcium [Ca2^+^]_i _was measured using the fluorescent Ca^2+ ^chelator Fura-2. As shown in Figure 1A, four days of TD (TD4) caused a significant increase in resting [Ca^2+^]_i_; [Ca^2+^]_i _was approximately 200 nM and 900 nM in control and TD cultures, respectively, suggesting that TD increased the influx of Ca^2+^. Since AMPARs are important mediators of Ca^2+ ^influx, we sought to determine whether TD affected AMPA-elicited Ca^2+ ^influx. Cells were incubated with a low-affinity fluorescent Ca^2+ ^probe Fluo-3 and challenged with a brief pulse (5 sec) of AMPA (30 μM). More than 95% of the monitored cells responded to AMPA stimulation and TD4 increased Fluo-3 fluorescence intensity (Figure 1B).

The analysis of Fluo-3 fluorescence imaging on a single cell indicated that AMPA-induced Ca^2+ ^influx peaked at 3-10 sec and returned to basal levels within 2 min (Figure 1C). To verify AMPA-induced Ca^2+ ^influx was mediated by AMPA receptors, after an initial AMPA pulse, neurons were exposed to a second AMPA pulse in the presence of a general AMPA receptor antagonist, GYKI. The AMPA-induced increase in [Ca^2+^]_i _was inhibited by GYKI, indicating the currents were mediated by AMPARs (Figure 1C). The specificity of AMPA-mediated response was further supported by the perfusion of GABAA receptor antagonist bicuculline plus Na^+ ^channel blocker tetrodotoxin which failed to block AMPA-increased [Ca^2+^]_i _(Figure 1E). Furthermore, the use of a calcium-free buffer eliminated an AMPA-triggered rise in [Ca^2+^]_i_, indicating the calcium response is dependent on extracellular Ca^2+ ^(data not shown). Thus, AMPA-mediated calcium elevations in cultured cells are primarily through Ca^2+^-permeable AMPARs. More importantly, TD4 drastically potentiated AMPA-elicited Ca^2+ ^influx (Figure 1C and 1D).

### TD inhibits GluR2 RNA editing in cortical neurons

**Figure 2:**
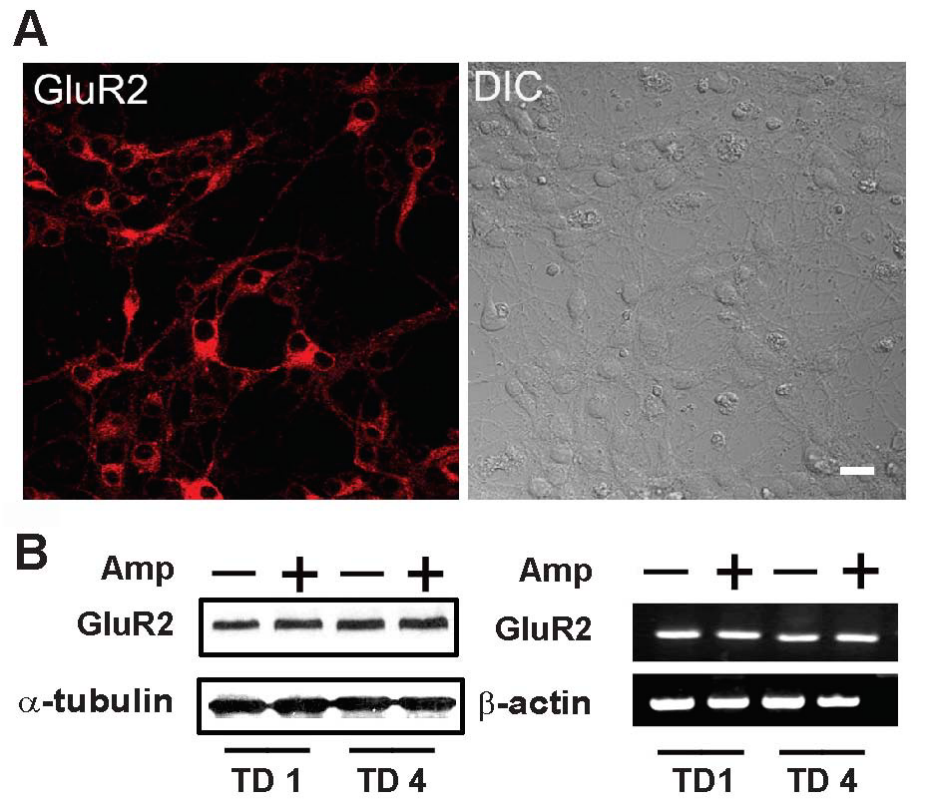
**The expression of GluR2 AMPA receptor in cultured cortical neurons**. **A**: The expression of GluR2 in cortical neurons of DIV7 was examined by immunofluorescence analysis (right panel). The DIC image was shown for comparison (left panel). **B**: Cortical neurons were treated with amprolium (Amp, 0 or 1 mmol/L) for 1 or 4 days. The expression of GluR2 was examined by immunoblotting analysis. The expression of α-tubulin served as a loading control. **C**: The mRNA levels of GluR2 were determined by RT-PCR. The expression of β-actin mRNA served as an internal control. The experiments were replicated three times.

**Figure 3:**
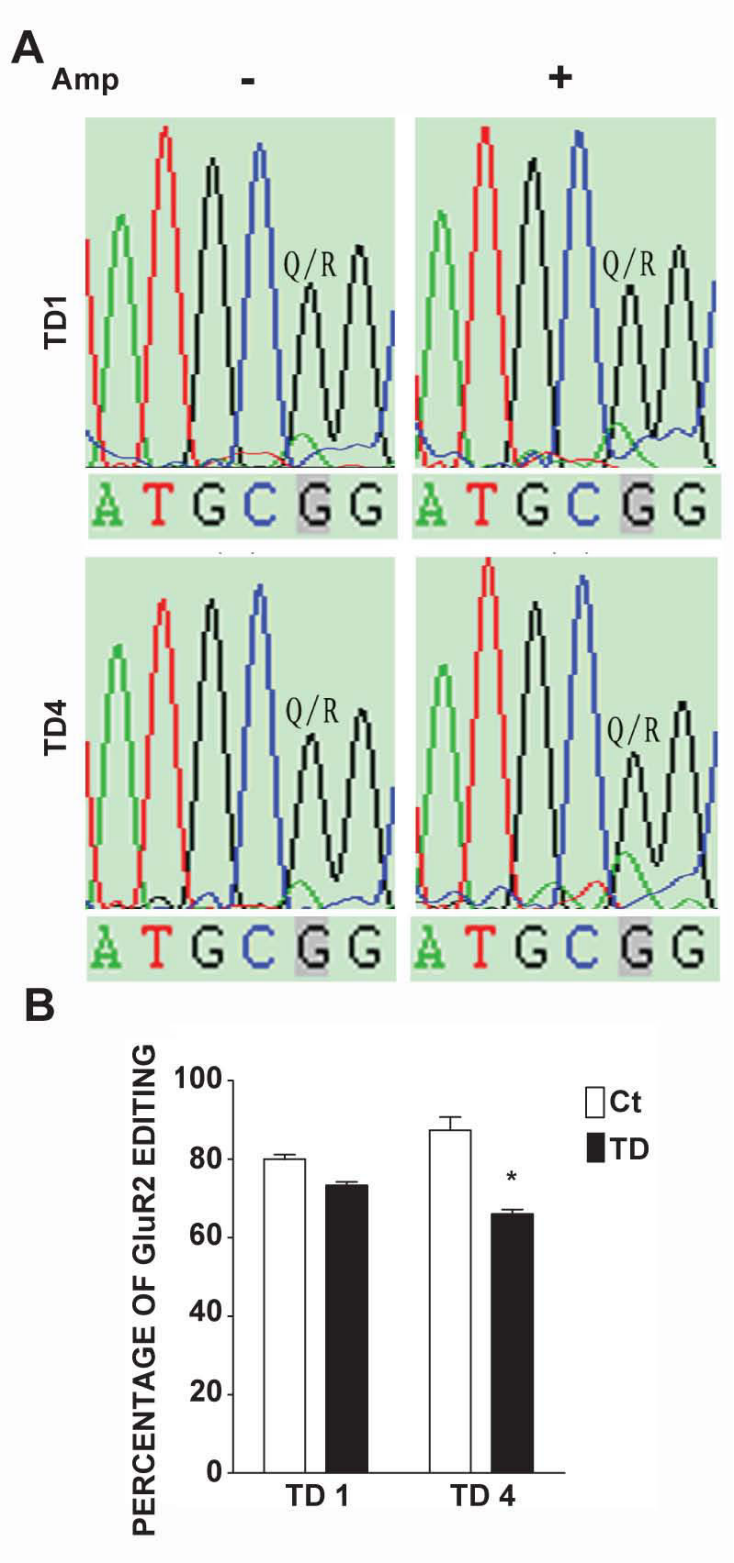
**Effects of TD on the editing efficiency of GluR2 mRNA**. **A**: Cortical neurons were treated with amprolium (Amp, 0 or 1 mmol/L) for 1 or 4 days (TD1 or TD4). Total RNA was isolated and PCR products of GluR2 were sequenced as described under the Materials and Methods. Sequence chromatograms of GluR2 transcripts were presented. Q/R indicates the glutamine/arginine editing position. The ratio of G (black) to A (green) reflects the editing efficiency. **B**: The Q/R site editing efficiency was calculated. The results were expressed as the mean ± SEM. *p <0.001. The experiments were replicated three times.

We sought to determine the mechanisms underlying TD-mediated alteration of Ca^2+^-permeable AMPARs. The GluR2 subtype of AMPARs regulates Ca^2+^-permeability. The immunocytochemical studies indicated that GluR2 was widely expressed in cultured cortical neurons (Figure 2A). TD did not alter either the protein or mRNA levels of GluR2 (Figure 2B and 2C). Since the glutamine/arginine (Q/R) site editing of GluR2 pre-mRNA controls the Ca^2+ ^permeability of AMPAR complexes, we investigated the effect of TD on Q/R site editing. Direct sequencing of the RT-PCR products demonstrated that there was a Q/R site editing of GluR2 mRNA in cultured cortical neurons (Figure 3A). We then determined the efficiency of editing by calculating the percentage of edited products. TD significantly decreased the efficiency of Q/R editing (Figure 3B).

### TD decreases ADAR2 expression and activity

**Figure 4:**
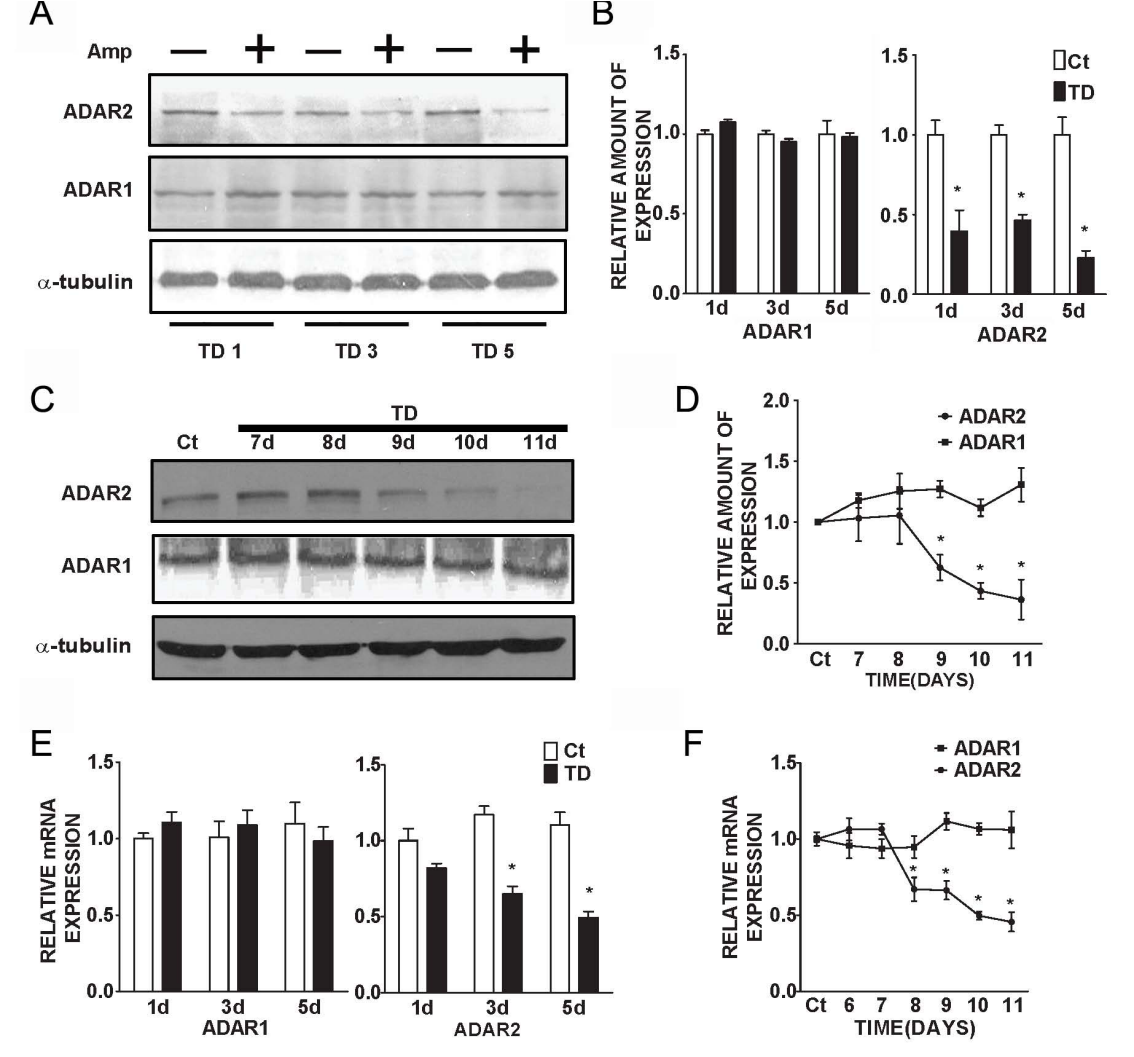
**The expression of ADAR2 in cultured cortical neurons**. **A**: Cortical neurons were treated with amprolium (Amp, 0 or 1 mmol/L) for 1, 3 or 5 days. The expression of ADAR1 and ADAR2 was examined by immunoblotting analysis. The expression of α-tubulin served as a loading control. **B**: The relative amounts of ADAR1 or ADAR2 protein were measured microdensitometrically and normalized to the expression of α-tubulin. **C**: TD was induced in mice as described under the Materials and Methods. At specified times after TD, the brain was removed (n = 5 for each treatment group). The expression of ADAR1 and ADAR2 was determined by immunoblotting analysis. **D**: The relative amounts of ADAR1 and ADAR2 protein in the brain were measured microdensitometrically and normalized to the expression of α-tubulin. **E**: Cortical neurons were treated with amprolium (Amp, 0 or 1 mmol/L) for 1, 3 or 5 days. The relative mRNA levels of ADAR1 and ADAR2 were quantified with real time PCR as described under the Materials and Methods. **F**: TD was induced in mice as described above. At specified times after TD, the brain was removed (n = 5 for each group). The relative mRNA levels of ADAR1 and ADAR2 were quantified with real time PCR. The results were expressed as the mean ± SEM. *p < 0.05. The experiments were replicated three times.

The Q/R editing of GluR2 is mediated by ADAR2 [37,38]. The decrease in RNA editing at Q/R sites suggests that TD may inhibit ADAR2 activity or expression. We examined the effect of TD on the expression of ADAR2 as well as ADAR2 self-editing activity in cultured cortical neurons. Immunoblotting analysis indicated that TD significantly decreased the expression of ADAR2, but not ADAR1 in cortical neurons (Figure 4A and 4B). A real-time PCR study showed that TD down-regulated mRNA levels of ADAR2, but not that of ADAR1 in cortical neurons (Figure 4E). Furthermore, TD in C57BL/6J mice also selectively reduced both mRNA and protein levels of ADAR2, but not that of ADAR1 (Figure 4C, 4D and 4F).

**Figure 5:**
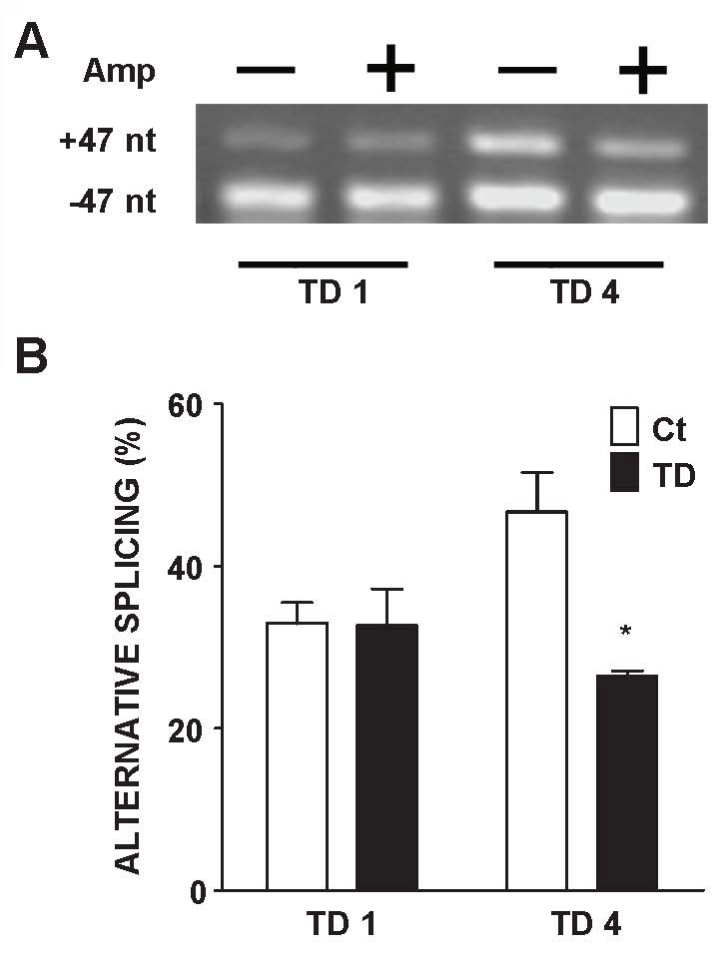
**Effects of TD on ADAR2 self-editing activity in cultured cortical neurons**. **A**: Cortical neurons were treated with amprolium (Amp, 0 or 1 mmol/L) for 1 or 4 days (TD1 or TD4). The expression of ADAR2 mRNA was analyzed by RT-PCR as described under the Materials and Methods. The products of alternative splicing of ADAR2 mRNA (+ 47 nt) were examined. **B: **The self-editing efficiency of ADAR2 mRNA was quantified by calculating the percentage of alternative splicing products (+47 nt). The results were expressed as the mean ± SEM. *p < 0.05. The experiments were replicated three times.

The self-editing of ADAR2 pre-mRNA has been used to evaluate ADAR2 activity [39]. A to I editing by ADAR2 within intron 4 creates an AI (= AG) 3' splice site that leads to alternatively spliced mRNA with a 47-nucleotide (nt) insert [40]. We examined the effect of TD on ADAR2 self-editing-dependent alternative splicing. As shown in Figure 5 TD significantly reduced the percentage of alternatively spliced products (+47 nt), indicating a decreased ADAR2 self-editing activity.

### Manipulation of ADAR2 expression alters AMPA-mediated Ca^2+ ^influx and TD-induced cytoxicity

**Figure 6:**
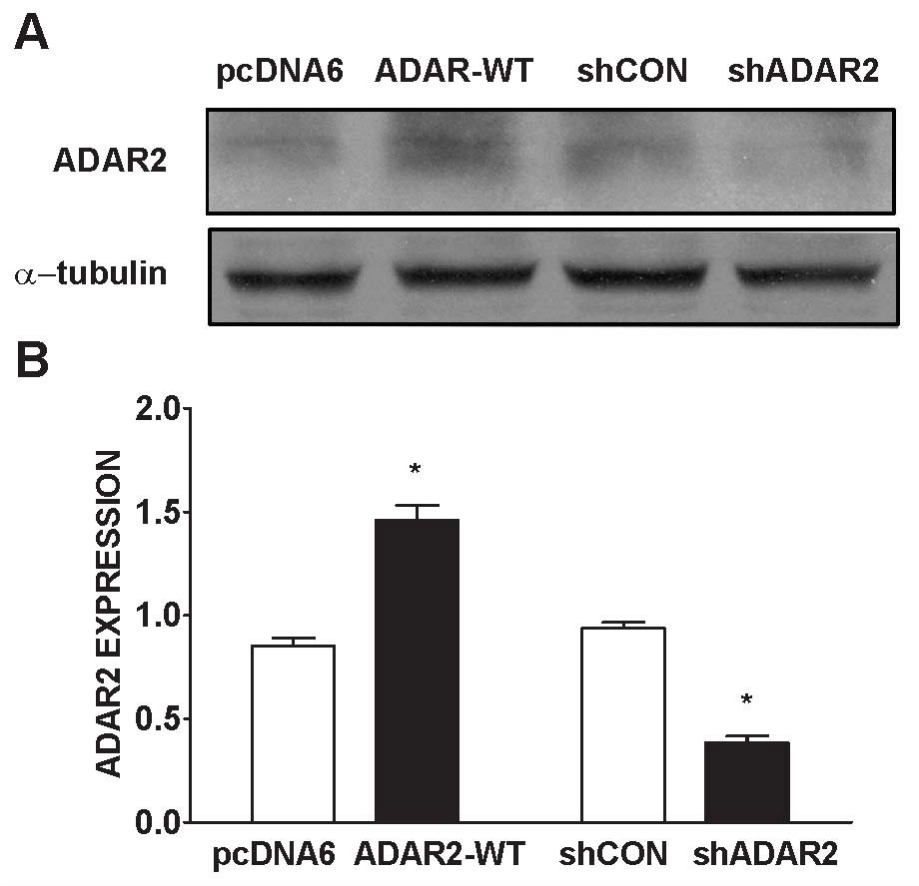
**The transfection of ADAR2 cDNA and shADAR2 RNA in cultured cortical neurons**. **A**. Cortical neurons were transfected with empty vector pcDNA6, pcDNA6 carrying wild-type ADAR2 cDNA, scramble short hairpin RNA (shCon) and shRNA for ADAR2 (shADAR2) along with a YFP construct for two days as described under the Materials and Methods. The expression of ADAR2 was determined by immunoblotting analysis. **B**: The expression of ADAR2 was quantified microdensitometrically and normalized to the expression of α-tubulin. The results were expressed as the mean ± SEM of three independent assays. *p < 0.05.

**Figure 7:**
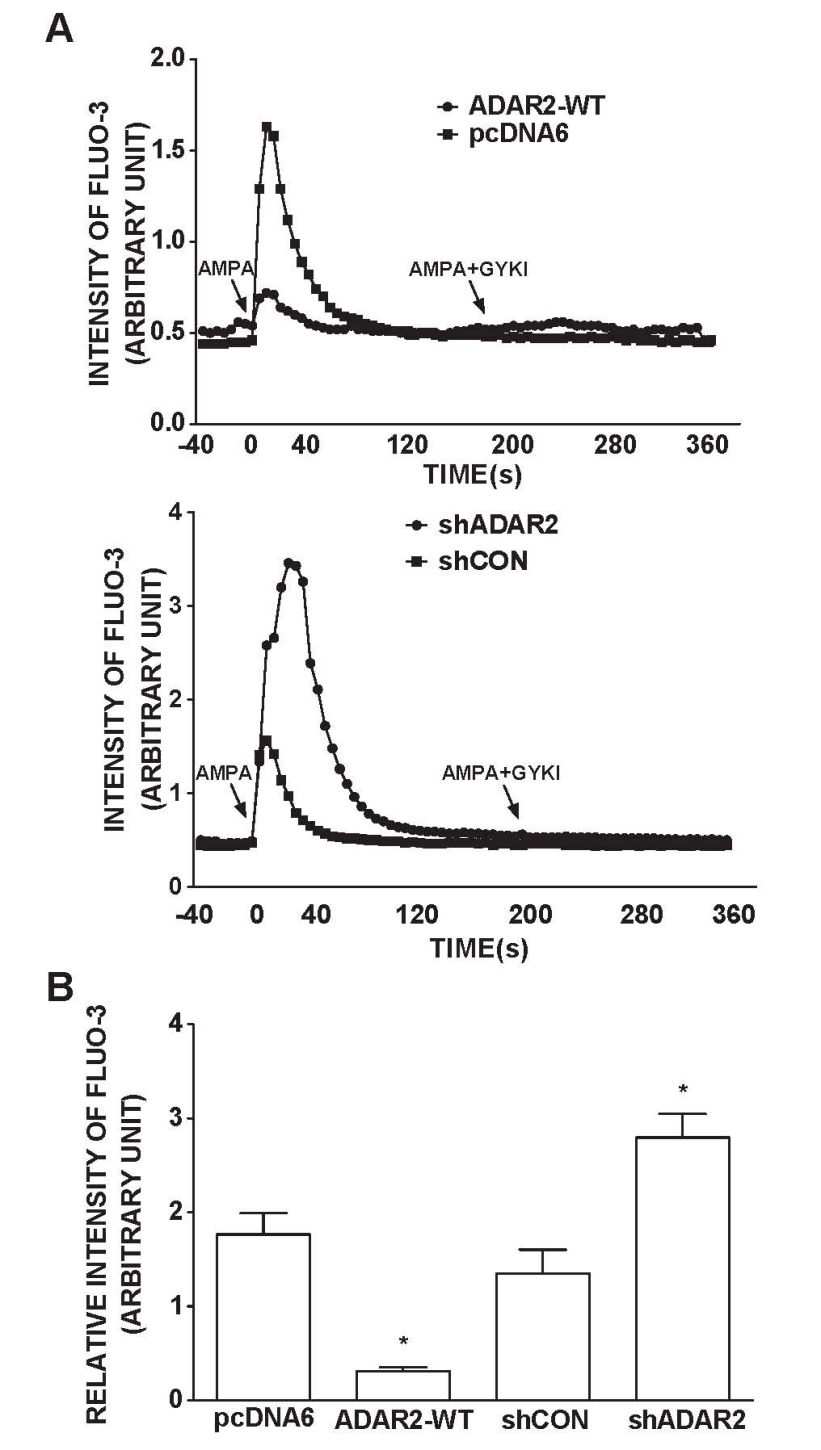
**Effects of ADAR2 over-expression or ADAR2 knock-down on AMPA-triggered [Ca2+]_i_**. **A**: Cortical neurons were transfected with empty vector pcDNA6, pcDNA6 carrying wild-type ADAR2 cDNA, scrambled short hairpin RNA (shCon) and shRNA for ADAR2 (shADAR2) along with a YFP construct for two days. After that, neurons were perfused with AMPA (30 μM) for 5 seconds. Two minutes after the first AMPA perfusion, neurons were subjected to a second AMPA perfusion with/without a general AMPAR antagonist GYKI (30 μM). [Ca^2+^]_i _was determined by single cells Fluo-3 fluorescence imaging as described under the Materials and Methods. **B**: The intensity of Fluo-3 fluorescence was quantified as described under the Materials and Methods. The results were calculated based on 75 cells. The results were the mean ± SEM from three independent assays. *p < 0.05.

**Figure 8:**
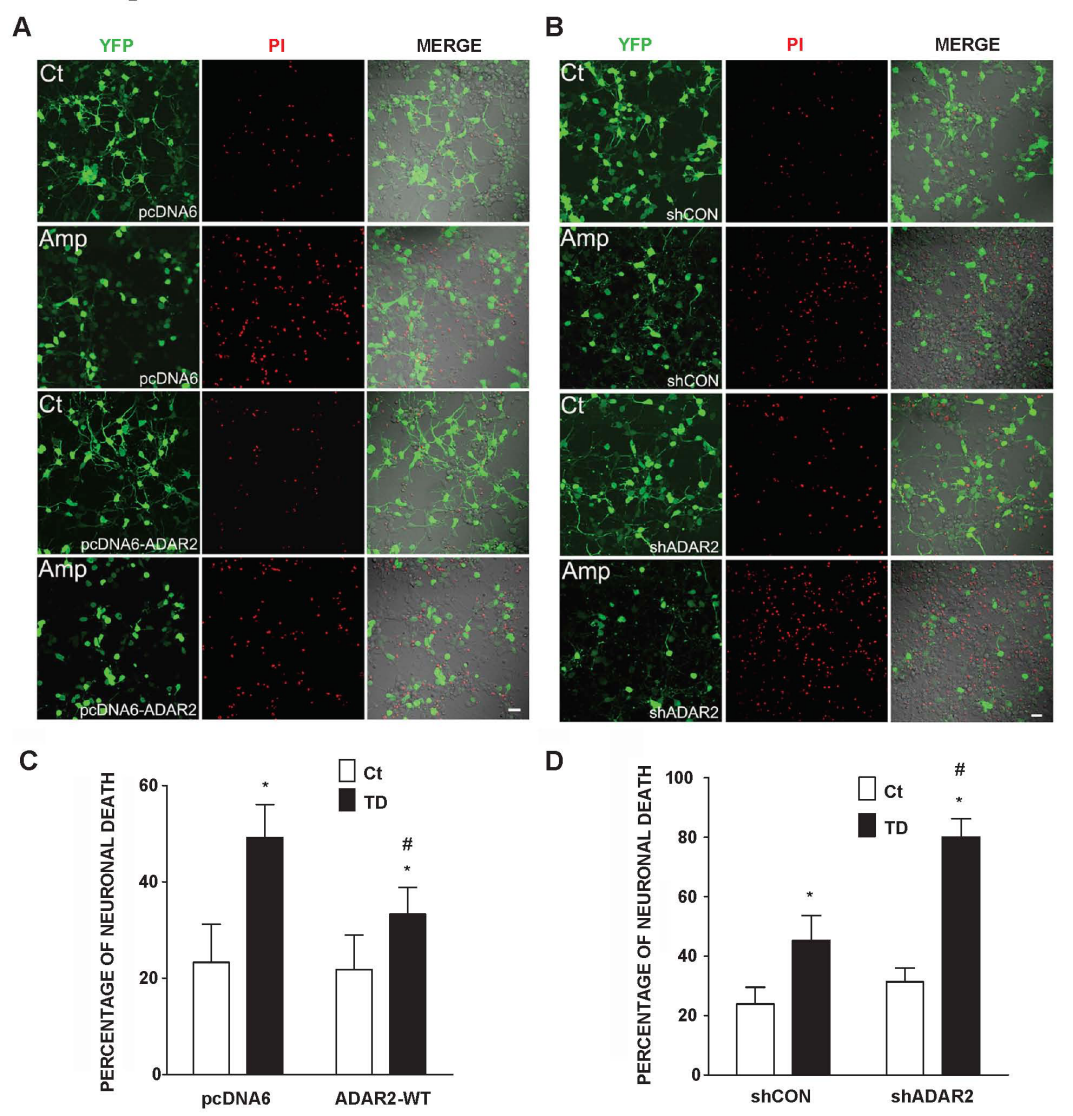
**Effects of ADAR2 over-expression or ADAR2 knock-down on TD-induced death of cortical neurons**. **A**: Cortical neurons were transfected with wild-type ADAR2 construct or pcDNA6 vector along with YFP construct and treated with amprolium (Amp, 0 or 1 mmol/L) for 4 days. The cytotoxicity was determined by propidium iodide (PI) staining (top panel). The percentage of PI-positive neurons in culture treated with wild-type ADAR2 or pcDNA6 was quantified (bottom panel). The results were expressed as the mean ± SEM of three independent assays. * signifies statistical differences from the controls (p < 0.05). # signifies statistical differences from the cultures treated with TD and pcDNA6 (p < 0.05). **B**: Cortical neurons were transfected with shADAR2 or shCON and treated with amprolium (Amp, 0 or 1 mmol/L) for 4 days. The cytotoxicity was determined by PI staining (top panel). The percentage of PI-positive cells in cultures treated with shADAR2 or shCON was quantified (bottom). The results were expressed as the mean ± SEM of three independent assays. * signifies statistical differences from the controls (p < 0.05). # signifies statistical differences from the cultures treated with TD and shCON (p < 0.05).

To further determine the role of ADAR2 in AMPA-mediated Ca^2+ ^influx and TD-induced cytoxicity, we manipulated the expression of ADAR2 in cortical neurons. We increased ADAR2 expression in cortical neurons by transfecting cells with a wild type ADAR2 cDNA, and decreased its expression by treating it with a short hairpin RNA (shRNA) of ADAR2. As shown in Figure 6 transfection of ADAR2 cDNA effectively increased ADAR2 expression, whereas shADAR2 decreased ADAR2 expression in cortical neurons. Over-expression of ADAR2 significantly inhibited an AMPA-mediated rise of [Ca^2+^]_i _in cortical neurons; contrarily, down-regulation of ADAR2 enhanced AMPA-mediated Ca^2+ ^influx, indicating the manipulation of ADAR2 expression was sufficient to alter Ca^2+ ^permeability of AMPARs channels (Figure 7). We further determined the role of ADAR2 in TD-induced neurotoxicity. As shown in Figure 8 over-expression of ADAR2 significantly ameliorated TD-induced death of cortical neurons; while down-regulation of ADAR2 enhanced TD-induced death of cortical neurons. These results suggest that ADAR2 may be involved in TD-induced death of cortical neurons.

## Discussion

In this study, we demonstrate for the first time that TD drastically increases [Ca^2+^]_i _in cortical neurons. The effect of TD is duration-dependent; TD treatment for 1 day (TD1) does not significantly alter [Ca^2+^]_i _whereas TD4 raises [Ca^2+^]_i _from approximately 200 nM to 900 nM. Consistent with the profile of alterations in [Ca^2+^]_i_, TD4, but not TD1, induces the death of cortical neurons (data not shown). The close correlation suggests an association between Ca^2+ ^influx and TD neurotoxicity. Further studies are necessary to confirm that TD-induced neurotoxicity is caused by the rise of [Ca^2+^]_i_. Ca^2+ ^ions are necessary for proper function and survival of neurons. Ca^2+ ^is a key regulator of synaptic plasticity. However in many pathological states, [Ca^2+^]_i _reaches critical levels leading to cell injury or death [21]. Ca^2+ ^overload can trigger several downstream lethal reactions, including nitrosative stress, oxidative stress, endoplasmic reticulum (ER) stress and mitochondrial dysfunction [15-17,41]. Ca^2+ ^is known to play a central role in excitotoxicity [42].

We have previously demonstrated that TD induces ER stress in animals and in cultured neurons [14]. ER stress refers to the accumulation of unfolded or misfolded proteins in the ER lumen, resulting in an overall decrease in translation, enhanced protein degradation and increased levels of ER chaperones, which consequently increase the protein folding capacity of the ER. Sustained ER stress ultimately leads to the apoptotic death of the cell [43,44]. A major cause of ER stress is the perturbation of calcium homeostasis [15-17]. Thiamine selectively diminished the reactive nitrogen species (RNS) and capacitative calcium entry (CCE) in the endoplasmic reticulum of fibroblasts [45]. Therefore, it is likely that TD-induced ER stress in neurons results from alterations in [Ca^2+^]_i_.

Ca^2+ ^levels are increased in aging neurons and the dysregulation of [Ca^2+^]_i _is implicated in neurodegeneration that occurs in AD [42]. β-amyloid protein (Aβ) stimulates Ca^2+ ^influx and raises [Ca^2+^]_i _in neurons, resulting in neuronal death [42]. TD in animals has been used to model some pathogenesis processes of AD. The current findings reveal that dysregulation of Ca^2+ ^may be another common feature for TD-mediated neuronal damage and neurodegeneration associated with AD.

Ca^2+ ^can gain entry into neurons through several mechanisms. Since TD increases glutamate levels in the brain, we investigate the effect of TD on glutamate receptor-regulated Ca^2+ ^influx. Our results indicate that TD drastically potentiates AMPA-mediated Ca^2+ ^influx. A recent study shows that Ca^2+^-permeable AMPARs are induced by *in vitro *traumatic mechanical injury in cortical neurons which results in increased [Ca^2+^]_i _[46]. A TD-induced increase in [Ca^2+^]_i _may be caused by the dysregulation of Ca^2+ ^-permeable AMPARs. GluR2 is a critical subunit in determining many of the major biophysical properties of AMPARs, including, but not limited to, Ca^2+ ^permeability, receptor kinetics, single-channel conductance and blockage by endogenous polyamines [47]. The great majority of AMPARs in the CNS exist as heteromers containing GluR2. AMPARs lacking GluR2 are permeable to Ca^2+ ^and Zn^2+^. Ca^2+ ^permeation through AMPARs is crucial in several forms of synaptic plasticity and cell death associated with neurological diseases and disorders. The subunit composition and Ca^2+ ^permeability of AMPARs are not static, but they are dynamically remodeled in a cell- and synapse-specific manner during development and in response to neuronal activity. The subunit composition and permeability of AMPARs are also remodeled by neuronal insults, such as seizures, ischemia, excitotoxicity, spinal cord injury and neurological diseases [e.g. AD, amyotrophic lateral sclerosis (ALS)] [48].

The changes in GluR2 properties may serve as a 'molecular switch' leading to the formation of Ca^2+^-permeable AMPARs and enhanced toxicity following neurological insults [49]. These changes arise not only from dysregulation of the expression of GluR2, but also because of RNA editing [50] and receptor trafficking [51]. Abnormal editing of GluR2 pre-mRNA at the Q/R site has been demonstrated to be associated with certain neurological insults such as ALS and brain ischemia [41,52]. A recent study indicates that inhibiting RNA editing of GluR2 at the Q/R site enhances the death of motor neurons through excitoxicity, whereas enhanced RNA editing reduces calcium permeability and protects motor neurons [52]. Furthermore, the expression of AMPARs with unedited GluR2 is highly toxic in cultured hippocampal neurons [53]. Interestingly, RNA editing at the Q/R site of GluR2 is involved in neuronal differentiation [29,54], supporting the notion that there is a physiological role of GluR2 RNA editing in the CNS development.

We demonstrate that TD does not affect GluR2 expression, but inhibits its RNA editing. The profile of TD-induced inhibition of GluR2 RNA editing is consistent with TD's effect on Ca^2+ ^influx; TD1 induces a modest inhibition of RNA editing, while TD4 produces much more inhibition (Figure 3). Therefore, TD increases the ratio of unedited GluR2 which is permeable to Ca^2+^.

The pre-mRNA editing of GluR2 at the Q/R site is regulated by a nuclear enzyme, ADAR2. ADAR2 knockout mice are fatal, indicating ADAR2 plays an essential role for survival [37]. Interestingly, TD selectively down-regulates ADAR2 expression without affecting ADAR1 *in vitro *and *in vivo*, indicating some specificity of TD's action. It has been shown that NF-κB regulates the expression of ADAR2 [55]and TD inhibits the transcription activity of NF-κB and AP-1 in neuronal cells [56]. Therefore, it is likely that TD down-regulates ADAR2 by modulating the activity of transcription factors. Generally, the pattern of TD-induced inhibition of ADAR2 expression is consistent with that of its interference with GluR2 RNA editing, i.e., longer TD exposure causes more inhibition. TD-induced down-regulation of ADAR2 is accompanied with a decrease in ADAR2 transcripts, suggesting that TD may affect either the transcription or mRNA stability of ADAR2. TD also inhibits ADAR2 self-editing which is indicated by a significant decrease in alternatively spliced products (+47 nt), suggesting that TD may also interfere with ADAR2 enzymatic activity.

## Conclusions

The contribution of ADAR2 in AMPA-mediated Ca^2+ ^influx in cortical neurons is confirmed by experiments that manipulate the expression levels of ADAR2 in cortical neurons. Over-expression of ADAR2 inhibits AMPA-mediated Ca^2+ ^influx, while knocking-down ADAR2 enhances AMPA-mediated Ca^2+ ^influx. In parallel, high expression of ADAR2 offers neuroprotection against TD-induced cytotoxicity, whereas down-regulation of ADAR2 exacerbated TD-induced death of cortical neurons. Similar to our finding, Peng et al., (2006) [41] show that forebrain ischemia in adult rats selectively reduces the expression of ADAR2 and disrupts RNA Q/R site editing of GluR2 in vulnerable neurons. Expression of exogenous ADAR2 or induction of endogenous ADAR2 expression protects neurons from forebrain ischemia insult [41]. Taken together, these results suggest TD-mediated inhibition of ADAR2 expression/activity causes a disruption of GluR2 RNA editing, resulting in increased Ca^2+ ^influx and elevated [Ca^2+^]_i_. Thus, ADAR2-dependent GluR2 Q/R site editing may determine vulnerability of neurons to TD.

## Material and methods

### Materials

Fura-2 was obtained from Invitrogen (Carlsbad, CA, USA). Fluo-3 was obtained from Calbiochem (EMD Chemicals, Inc. Gibbstown, NJ, USA). Anti-GluR2 antibody was obtained from Chemicon (Temecula, Ca, USA). Anti-ADAR2 and ADAR1 antibodies were obtained from Santa Cruz Biotech (Santa Cruz, CA, USA). The secondary Texas Red conjugated donkey anti-rabbit IgG for GluR2 and FITC-conjugated donkey anti-Goat IgG for ADAR2 were obtained from Jackson ImmunoResearch Laboratories (West Grove, PA, USA). All other chemicals were obtained from Sigma Chemical Co. (St. Louis, MO, USA).

### Culture of cortical neurons

Primary cultures of cortical neurons were generated using a previously described method [57,58]. Briefly, the 14 day C57BL/6J mouse embryos were decapitated and cerebra were removed. The cerebra were minced with a sterile razor blade and suspended in 10 ml of trypsin solution (0.025%) at 37°C. After incubation for 15 min, an equal volume of a solution containing DNase (130 Kunitz units/ml) and trypsin inhibitor (0.75 mg/ml) was added, and the tissue was sedimented by a brief (5 s) centrifugation. The tissue was dissociated by trituration, and the cell suspension was mixed with 4% bovine serum albumin and centrifuged. The cell pellet was resuspended in Neurobasal/B27 medium containing 2% B27 and 1 mmol/L glutamine (Invitrogen Corporation, Carlsbad, CA, USA), 100 units/ml penicillin and 100 μg/ml streptomycin (Gibco Inc.; Los Angeles, CA, USA). Cells were plated into poly-D-lysine (50 μg/ml) coated cell culture wells or dishes, and maintained at 37°C in a humidified environment containing 5% CO_2 _for 7 days *in vitro *(DIV7).

### Induction of thiamine deficiency

To induce TD *in vitro*, cortical neurons of DIV7 were treated with amprolium (1 mmol/L) for indicated times [8,14,36]. Amprolium is a competitive inhibitor of thiamine transport and effectively depletes intracellular thiamine. The animal TD model has been previously described [1]. Briefly, adult male C57BL/6J mice (20-25 g) were housed in a controlled environment (one mouse/cage at 23°C and 53% humidity). The animals were fed with either a control diet or a thiamine deficient diet (ICN Nutrition Biomedicals, Cleveland, OH, USA) *ad libitum*. TD animals also received a daily i.p. injection of a thiamine antagonist, pyrithiamine hydrobromide (5 μg/10 g body weight), while control animals were injected with saline. Pyrithiamine is a potent inhibitor of thiamine pyrophosphokinase and blocks the synthesis of thiamine diphosphate (TDP).

### Measurement of propidium iodide (PI) uptake

Cytotoxicity was analyzed by propidium iodide (PI) uptake. Neuronal cultures were incubated with PI (20 μg/ml) for 15 min. The fluorescence signals were detected by a Zeiss LSM 510 confocal system (Carl Zeiss MicroImaging, Inc., Thornwood, New York) on an inverted microscope. The excitation and emission wavelengths were 490 and 615 nm, respectively.

### Measurements of intracellular free calcium

The concentrations of intracellular free calcium [Ca^2+^]_i _were measured using Fura-2/acetoxymethyl ester (Fura-2/AM) as described previously with some modifications [59]. The measurement was performed in 96-well microtiter plates. Briefly, cells were incubated in Hank's solution containing Fura-2/AM (5 μm) at 37°C for 45 min. Cells were alternatively excited with 340 and 380 nm wavelengths, and fluorescence signals were detected with the FlexStation (Molecular Devices, Wokingham, UK). Fluorescence ratios were converted to [Ca^2+^]_i _by using the equation of [Ca^2+^]_i _= Kd*(F-F_min_)/(F_max_-F) [60] with apparent dissociation constant *Kd *= 0.224 mM. The *R*_min _and *R*_max _were determined by equilibrating [Ca^2+^]_i _with "calcium-free" (0 mM Ca^2+^/10 mM EGTA) or high Ca^2+^(10 mM Ca^2+ ^in Hepes), respectively.

[Ca^2+^]_i _levels were also quantified by single cell Fluo-3 fluorescence image as described previously [61] with some modifications. Briefly, cells were loaded with Fluo-3/AM (2 μM, Calbiochem) in Ringer solution at 37°C for 45 min, then transferred to a perfusion chamber and perfused with calcium buffer or calcium-free buffer. Cells were perfused at 1 ml/min at room temperature using a peristaltic pump. The calcium image was recorded with a confocal laser scanning microscope (Carl Zeiss Axiovert 200 LSM 510, Jena, Germany). A field including 15-20 cells was selected. Successive images of 512×512 pixels were collected at intervals of 1.97 s with a 40× oil-immersion objective and each series of images consisted of 300 sections. Fluo-3 fluorescence was excited at 488 nm and emitted light was measured at 530 nm. The intensity of Fluo-3 fluorescence intensity represented [Ca^2+^]_i_. The quantification of Fluo-3 fluorescence intensity was performed using the software Rel.3.2 (Carl Zeiss MicroImaging, Inc., Thornwood, New York).

### Immunocytochemistry

Neuronal cultures on cover slips were treated with amprolium for indicated times and fixed with 4% paraformaldehyde. The cultures were incubated with primary antibodies at 4°C overnight at the following concentrations: Anti-GluR2 antibody, 0.5 μg/ml; anti-ADAR2 antibody, 1:500. This was followed by secondary antibody treatment (Texas Red conjugated donkey anti-rabbit IgG or FITC-conjugated donkey anti-Goat IgG at a dilution of 1:100). Fluorescent images were visualized with a Zeiss LSM 510 confocal system (Carl Zeiss MicroImaging, Inc., Thornwood, NY) using a 40× oil-immersion objective lens. Differential interference contrast (DIC) images were also taken for comparison.

### Immunoblotting

The immunoblotting procedure has been previously described [14]. Briefly, proteins were separated using SDS-polyacrylamide gels and transferred to nitrocellulose membranes. The membranes were blocked with 5% nonfat dry milk or 5% BSA in 0.01 M PBS (pH 7.4) and 0.05% Tween-20 (TPBS) at room temperature for 1 h. Subsequently, the membranes were incubated with primary antibodies directed against target proteins overnight at 4°C. The final dilutions for primary antibodies were: anti-GluR2 1:2,000; anti-ADAR2, 1:1,000; anti-ADAR1, 1:1000). The blots were washed, incubated with a secondary antibody conjugated to horseradish peroxidase (Amersham, Arlington Heights, IL, USA), diluted at 1:5,000 in TPBS for 2 h, and visualized by the enhanced chemiluminescence (ECL) method (Amersham). The expression of specific proteins was quantified with the software of Quantity One (Bio-Rad Laboratories, Hercules, CA, USA).

### ADAR2 self-editing assay and Q/R editing assay

Total RNA was isolated from cultured neurons with TRIzol reagent (Invitrogen Corporation, Carlsbad, CA, USA) followed by digestion with RNase-free DNase I (Roche Diagnostics Corp. Indianapolis, IN, USA) to eliminate possible DNA contamination. First-strand cDNA was synthesized by moloney murine leukemia virus reverse transcriptase and Oligo dT primers (Promega Corporation, Madison, WI, USA) [40].

cDNA (100 ng) was subjected to PCR in a 20 μL reaction solution containing 2× Taq Premixure buffer (Tiangen Biotech, Beijing, China) and 20 μM primer. The PCR amplification protocol was 95°C for 5 min, followed by 40 cycles of 95°C for 30 s, 55°C for 30 s, and 72°C for 15 s and finished with a final extension of 72°C for 10 min. PCR primers used in this study are as follows: ADAR2 forward, 5'-GCCAGTCAAGAAGCCCTCA-3'; ADAR2 reverse, 5'-TGTCCAGATTGCGGTTTT-3'; GluR2 forward, 5'-TTGAAGGGAATGAGCGTTAT-3'; GluR2 reverse, 5'-GCCGTGTAGGAGGAGATG-3'. The PCR products of ADAR2 were run on a 1% agarose gel and visualized by the Dolphin-Scan image system. The unedited PCR products of ADAR2 were 102 bp, and the edited PCR products of ADAR2 were 149 bp. The editing efficiency was quantified using Dolphin-1 D software (Wealtec, Sparks, NV, USA), and the percentage of ADAR2 self-editing was calculated. The PCR products of GluR2 were sequenced and analyzed for its Q/R site editing [40].

### Real-time PCR

Real-time quantitative PCR was conducted with ABI Prism 7500 Sequence Detection System according to the instruction of the manufacturer (Applied Biosystems, Foster City, CA, USA). RT-PCR primers used in this study are as follows: GluR2 forward, 5'-CCATGAAAGTGGGAGGTAACTTG-3'; GluR2 reverse, 5'-AAGCCCCTGCTCGTTCAGT-3'; ADAR2 forward, 5'-TGTAAGCACGCGCTGTACTGT-3'; ADAR2 reverse, 5'-GACTCGTGGTATGTGGTAGGCTTAG-3'; β-actin forward, 5'-GATCATTGCTCCTCCTGAGC-3'; β-actin reverse, 5'-ACTCCTGCTTGCTGATCCAC-3'.

For relative comparison of each gene, we analyzed the *Ct *value of real-time PCR data with the ΔΔCt method normalized by an endogenous control (β-actin) [62].

### Plasmids and cell transfection

pcDNA6, pcDNA6-myc-ADAR2, Lentivirus-shADAR2 RNA (5'-GCAGCTGAACGAGATCAAACC-3'), Lentivirus-scramble RNA (5'-GATGTTGTCAACGACTAGTTT-3') and yellow fluorescence protein (YFP) were provided by Dr. Yong Liu (Institute for Nutritional Sciences, Shanghai, China). Cell transfection was performed using a Mouse Neuron Nucleofection kit (Amaxa, Koelh, Germany) [14]. Briefly, 2.5 × 10^6 ^cortical neurons were centrifuged and resuspended in 100 μL transfection buffer. One microgram of plasmids (pcDNA6-myc-ADAR2, pcDNA6, YFP, lentivirus-shADAR2 or Lentivirus-scramble RNA) together with one microgram of YFP was added to the neurons; the transfection was performed using an Amaxa Nucleofection apparatus. After the transfection, cells were transferred to cell culture dishes and grown in Neurobasal/B-27 media. The transfection efficiency was approximately 70% which was determined by YFP-positive cells.

### Statistical analysis

Data were presented as means ± SEM. Differences among treatment groups were assessed by ANOVA followed by Student-Newman-Keuls analyses. Differences in which *p *was less than 0.05 were considered statistically significant.

## Competing interests

The authors declare that they have no competing interests.

## Authors' contributions

SL, GY, YY, YL, LZ, JX, XZ and ZF carried out all of the experiments. CF carried out image analysis. ZK, JL, YL, YW and SL participated in the design of the study and the writing of the manuscript. All authors read and approved the final manuscript.
